# The lactate shuttle in ageing: a metabolic bridge between muscle fatigue and brain resilience

**DOI:** 10.3389/fphys.2026.1823430

**Published:** 2026-05-29

**Authors:** Yu Zhang, Wenyi Yang, Chunlan Tian

**Affiliations:** 1 School of Physical Education, Shandong University, Jinan, China; 2 Binzhou City Sports Bureau, Binzhou, China; 3 College of Physical Education Science, Qufu Normal University, Qufu, China

**Keywords:** aging, blood-brainbarrier, exercise, GPR81 receptor, histone lactylation, lactate, lactate shuttle, neurodegenerative diseases

## Abstract

Traditionally, lactate was considered a glycolytic byproduct that causes muscle fatigue, but now its biological role is undergoing a significant paradigm shift. Emerging evidence suggests that lactate acts as an inter-organ metabolic and signaling mediator linking exercise-induced peripheral metabolic stress to central nervous system adaptation. This review explores how exercise drives lactate pulses and delivers them to the brain through the circulatory system and blood-brain barrier (BBB). Lactate has a dual function in the brain, serving not only as the preferred energy substrate for active neurons but also as a core signaling molecule. Through pathways involving G protein coupled receptor 81 (GPR81) and histone lactylation, lactate regulates neuroplasticity, cerebrovascular function, neuroinflammation, and antioxidant defense, thereby establishing cognitive resilience. During aging, multiple components of this proposed lactate signaling axis may become compromised, including skeletal muscle lactate production, circulatory and blood-brain barrier transport, and cellular responsiveness within the brain. Such multi-level impairment may contribute to neuromuscular co-aging and may increase vulnerability to neurodegenerative disorders, including Alzheimer’s disease. Ultimately, we explored the translational potential of restoring the lactate signaling axis through multimodal strategies to promote healthy aging, including precise exercise prescriptions, GPR81 targeted therapy, metabolic interventions, and biomarker development. This review aims to combine metabolic science with evidence of neuroaging, providing a new theoretical framework for determining the primacy of exercise-driven brain health and advancing anti-aging interventions.

## 1 Introduction

Within the conventional framework of exercise physiology and metabolic science, lactate has long been regarded as the end-product of glycolysis in skeletal muscle under hypoxic conditions—a metabolic waste responsible for muscle soreness, fatigue, and decreased performance (Hargreaves and Spriet, 2020). This classical perspective prevailed for decades, positioning lactate as a passive end product in energy metabolic pathways. Nevertheless, a series of discoveries has significantly redefined our understanding of lactate’s physiological functions, facilitating a significant paradigm shift. The initial catalyst was the proposal and development of the lactate shuttle theory, which explained lactate as an active energy carrier, steadily transported and used between cells and organs via monocarboxylate transporters (MCTs), forming a dynamic metabolic cycle (Brooks, 2018).

More importantly, lactate has been reconceptualized as a signaling molecule with broad biological activity. Its role within the brain is notably striking: lactate is not only a favored energy substrate for neurons during active states, within the astrocyte-neuron lactate shuttle framework (Magistretti and Allaman, 2018), but also a core signal that directly modulates neuroplasticity, cerebral blood flow, and neuroinflammation via its specific receptor G-protein coupled receptor 81 (GPR81)/Hydroxycarboxylic Acid Receptor 1 (HCAR1) (Colucci et al., 2023). The current discovery of lactate as a precursor for histone lactylation has further expanded its roles into the range of epigenetic regulation, enabling metabolic state to directly influence gene expression (Zhang et al., 2019).

Taken together, these findings support the view that exercise-derived lactate may serve as an important metabolic and signaling intermediary linking peripheral muscle activity to central brain adaptation. Repeated exercise may generate recurrent lactate pulses that contribute to brain adaptation by supporting energy metabolism and engaging signaling pathways linked to neuroplasticity, angiogenesis, and inflammatory regulation (Zhu et al., 2025). Nevertheless, components of this integrative lactate signaling framework appear to be progressively impaired during aging. Sarcopenia causes an impaired signal source (Horlem et al., 2025); circulatory and Blood-Brain Barrier (BBB) dysfunction impede signal transmission (Mayorga-Weber et al., 2025); and the brain’s internal capacity to detect and respond to the lactate signal concurrently declines (Acevedo et al., 2023). This multi-level dysfunction may weaken exercise-related neuroprotective responses, contribute to neuromuscular co-aging, and intersect with pathogenic processes involved in neurodegenerative diseases such as Alzheimer’s disease (Liu and Zhou, 2024; Wang MY. et al., 2024).

Hence, this review seeks to explore the evolving role of the lactate shuttle during aging. In this review, we define the “lactate signaling axis” as an integrative framework rather than a fully resolved linear pathway. Some components of this framework, such as lactate transport and neuronal utilization, are well supported experimentally, whereas others, particularly their causal roles in aging-related neurodegeneration, remain emerging. We therefore distinguish throughout the manuscript between established findings, plausible mechanistic extensions, and hypothesis-generating interpretations.

## 2 The exercise-initiated lactate dialogue

**Figure 1:**
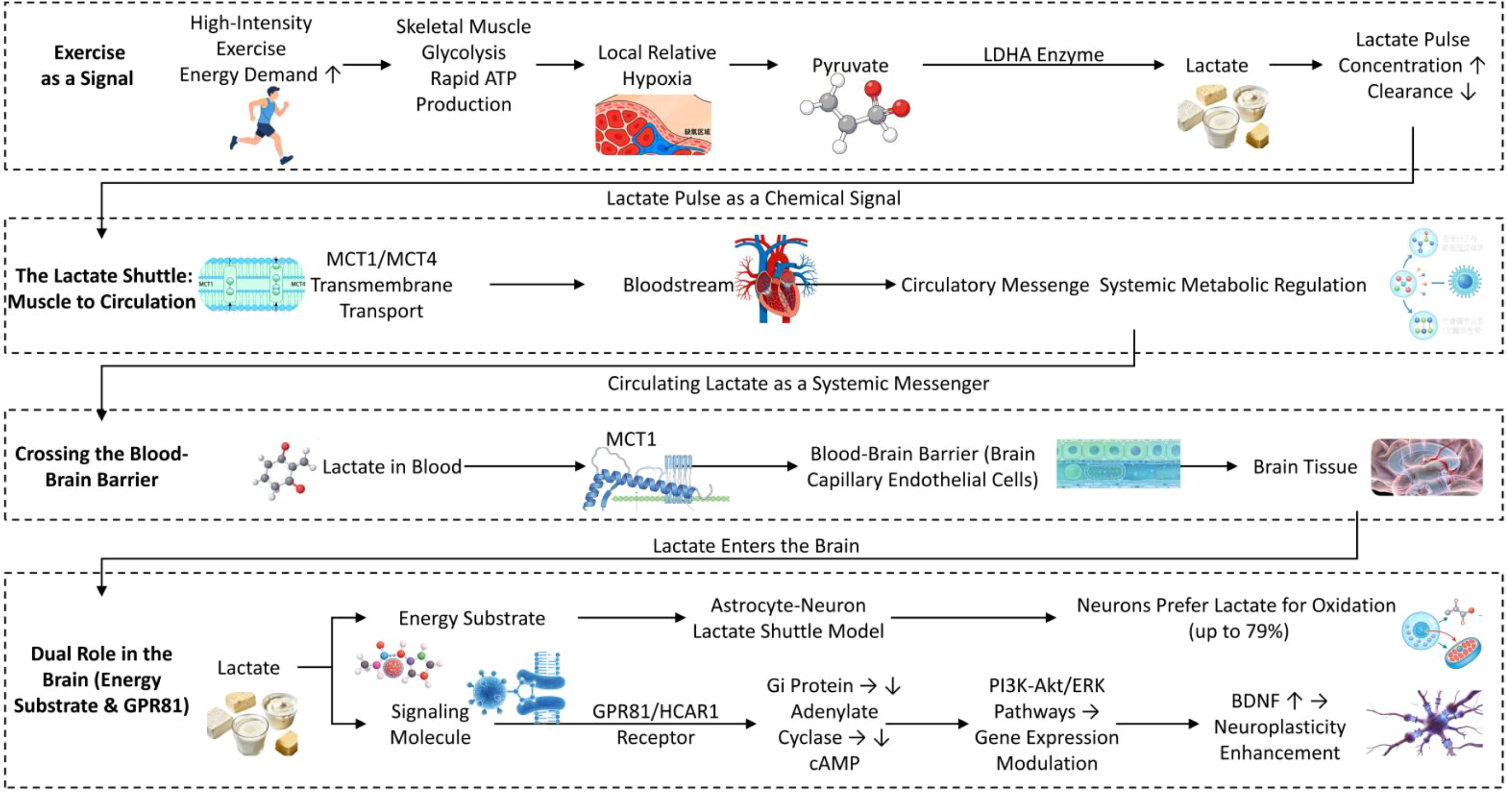
The lactate dialogue triggered by exercise represents a mechanistic pathway from muscle contraction to brain adaptation. This figure summarizes the proposed sequence by which exercise-induced lactate links skeletal muscle activity to brain adaptation. During high-intensity exercise, glycolytic flux increases in working muscle, leading to lactate production and transient release into the circulation through monocarboxylate transporters (MCTs). Circulating lactate then crosses the blood-brain barrier, mainly via MCT1, and enters the brain. Within neural tissue, lactate may serve both as an oxidative substrate for active cells and as a signaling molecule acting through GPR81/HCAR1-related pathways. Together, these processes provide a mechanistic framework through which acute exercise-related metabolic signals may influence neuroplasticity and brain function. BBB, blood-brain barrier; BDNF, brain-derived neurotrophic factor; GPR81, G protein-coupled receptor 81; HCAR1, hydroxycarboxylic acid receptor 1; LDHA, lactate dehydrogenase A; MCT, monocarboxylate transporter.

The physiological demands of physical activity initiate the production of lactate, transforming a simple metabolic byproduct into the primary language through which muscles will subsequently communicate with distal organs. This initial step—the generation of a lactate pulse—is therefore not merely a local event, but the fundamental trigger that sets the entire inter-organ dialogue in motion. **Figure 1** illustrates the proposed sequence through which exercise-induced lactate signaling links muscle contraction to brain adaptation.

### 2.1 Exercise as a signal trigger: the physiological process generating lactate pulses

Exercise, particularly when performed above or near the lactate threshold, is a major physiological stimulus for transient systemic lactate elevation (Hargreaves and Spriet, 2020; Spriet, 2022). During exercise, skeletal muscle relies heavily on glycolysis to rapidly generate adenosine triphosphate (ATP) in response to sharply increased energy demand (Hargreaves and Spriet, 2020; Spriet, 2022). Under conditions of adequate oxygen supply, the glycolytic product pyruvate penetrates mitochondria for further oxidation. Nevertheless, under local relative hypoxia driven by high-intensity exercise, pyruvate is promptly converted into lactate in the cytoplasm by the enzyme lactate dehydrogenase A (LDHA) (Wang and Zhu, 2025). The resulting lactate is exported from working muscle into the bloodstream, generating a transient lactate pulse characterized by a rise in circulating concentration followed by a clearance phase whose magnitude and duration depend on exercise intensity, training status, and metabolic fitness (Huang et al., 2025).

Rather than representing a passive metabolic byproduct, this transient lactate elevation is increasingly viewed as a context-dependent adaptive signal. Evidence indicates that lactate actively participates in intercellular communication as a signaling molecule and contributes to exercise-induced multisystem adaptations (Nalbandian and Takeda, 2016). Notably, in the brain, exercise-derived lactate is increasingly recognized as a signaling molecule that may transiently influence neural function. By functioning as both an oxidative substrate and a signaling mediator across neural cell types, lactate may contribute to the metabolic component of the exercise–brain connection, although it acts alongside other exercise-induced factors rather than in isolation (Zhu et al., 2025).

Hence, the lactate pulse caused by exercise holds significant systemic physiological significance. As a circulating metabolic messenger, it exerts autocrine, paracrine, and endocrine effects, impacting the distribution of energy substrates and whole-body metabolic regulation (Brooks, 2018). In essence, the lactate pulse translates the contractile state of muscles in real-time into a chemical signal perceivable by numerous organs throughout the body, notably the brain, providing a key signal linking the body’s metabolic balance, stress adaptation, and functional improvement.

### 2.2 The lactate shuttle journey from muscle to circulation

Following its generation within myofibers, lactate does not simply accumulate locally but is dynamically exchanged between intracellular, interstitial, and circulatory compartments. Instead, it promptly penetrates the bloodstream via an effective transmembrane transport system, initiating its role as a circulating messenger. This mechanism is mainly induced by a protein family referred to as MCTs (Seyedi et al., 2024). Intramuscular lactate handling depends on the balance between glycolytic production, mitochondrial utilization, and transporter-mediated efflux, with MCTs serving as key regulators of lactate exchange across the sarcolemma (Seyedi et al., 2024). Notably, MCT1, encoded by the SLC16A1 gene, is responsible for the co-transport of lactate and hydrogen ions across the cell membrane, hence directly impacting intracellular pH and lactate accumulation kinetics (Maculewicz et al., 2025). In skeletal muscle, MCT1 is typically associated with oxidative lactate uptake, whereas MCT4 is more closely linked to lactate efflux from highly glycolytic fibers (Bonen, 2001).

In various tissues, such as muscle and heart, lactate crosses the plasma membrane through stereospecific and pH-dependent MCT systems. Transcripts for MCT1 and MCT4 have been identified in both rat and human skeletal muscle and heart (Bonen, 2001). The MCT family (especially the most studied MCT1-MCT4 subtypes) is extensively expressed in numerous tissues, playing a critical role in mediating proton-coupled transmembrane transport of monocarboxylates, such as lactate (Sun et al., 2020).

Once in the bloodstream, lactate functions as a systemic messenger, being promptly transported to different distal organs, comprising the brain. The swift transport and ensuing metabolism of lactate are vital for sustaining energy homeostasis as the body responds to metabolic challenges, such as high-intensity exercise (Brooks, 2018). Hence, circulating lactate is more than just a carrier of metabolic products; it is a critical signaling molecule capable of conveying immediate information about energy demand, supporting exercise-related cognitive enhancement (Xue et al., 2022). Through this effective shuttle journey, lactate improves interorgan energy allocation and signal communication, playing a central physiological role. Within skeletal muscle itself, lactate also participates in redox balancing, substrate redistribution, and training-related remodeling, indicating that muscle is not only the source of lactate but also an active site of lactate signaling and reuse (Li et al., 2022; Zhang et al., 2024).

### 2.3 Crossing the BBB: the key role of MCT transporters

For lactate to enter the brain tissue from the circulation and exert its functions, it must cross the extremely selective BBB, which is composed of closely connected endothelial cells (Lochhead et al., 2020; Zhu et al., 2025). This critical step is mainly induced by members of the monocarboxylate transporter family. In the central nervous system (CNS), MCT1 is extensively expressed on brain capillary endothelial cells and astrocytes, while MCT2 is mainly uncovered on neurons, together forming the key network for lactate transport across cell membranes (Pierre and Pellerin, 2005). Literature indicates that MCT1 is the primary protein inducing the transport of lactate across the BBB through brain capillary endothelial cells, and its function is vital in lactate entry from the blood into the brain (Liu et al., 2016).

The activity and expression of MCTs are not static, but are exactly modulated by physiological state and age. Exercise, as a potent stimulus, can increase MCT1 protein content in human skeletal muscle, and MCT4 abundance may also adaptively change in response to training according to metabolic demands and training status (Benítez-Muñoz et al., 2024). Nevertheless, this transport system declines in function during aging. For example, in the corpus callosum, aging is linked to reduced glucose levels and lactate accumulation, alongside decreased MCT1 expression, hindering lactate transport and oxidative metabolism, stressing that age-associated metabolic modifications involve changes in monocarboxylate transporter function (Mayorga-Weber et al., 2025).

More critically, aging itself causes a general impairment of BBB function, which may improve the development of NDs, such as AD (Cunha et al., 2024). The degeneration of BBB structure and function, integrated with the downregulation of MCT expression on its surface, jointly impairs the effectiveness with which lactate—a vital energy source and metabolic signal for the brain—is transported to the CNS (Wu et al., 2023). Hence, MCT-induced lactate transport across the BBB is not only fundamental for sustaining cerebral energy supply, but also a decisive link in the successful activation of brain adaptive responses by the lactate signaling axis. Its dysregulation during aging carries vital pathophysiological implications.

### 2.4 The dual role in the brain: energy substrate and GPR81 receptor-induced signaling

Once lactate enters the brain, it may exert dual and complementary actions by serving as an oxidative substrate for active neural cells and as a signaling molecule that modulates cell-specific adaptive responses.

As an energy substrate, lactate offers an effective and promptly available oxidative fuel for active neurons. a key component of this mechanism is the astrocyte-neuron lactate shuttle model. In this model, astrocytes generate lactate through glycolysis, which is then discharged into the extracellular space through MCTs and then taken up by adjacent, extremely active neurons to be oxidized in mitochondria for energy (Magistretti and Allaman, 2018). Experimental evidence strongly supports the neuronal preference for lactate metabolism. In main neuronal cultures, when equimolar concentrations of glucose and lactate are present, lactate contributes up to 79% to neuronal oxidative metabolism, far exceeding the 21% contribution from glucose, manifestly determining that neurons preferentially use lactate as their main oxidative substrate (Bouzier-Sore et al., 2003).

In addition to its metabolic role, lactate may exert non-metabolic effects through GPR81/HCAR1-dependent as well as receptor-independent pathways (Vaccari-Cardoso et al., 2023). This G protein-coupled receptor is extensively expressed and functionally active in the mammalian brain, allowing lactate to function as a volume-transmitted neurochemical signal (Morland et al., 2015). Relevant target cells include neurons, astrocytes, endothelial cells, and possibly perivascular cell populations, suggesting that lactate signaling may influence the brain through multicellular rather than purely neuron-centered mechanisms (Lauritzen et al., 2014; Lee et al., 2022). Upon binding to GPR81, lactate functions as a metabolic sensor, activating downstream signaling cascades. The main pathway involves Gi-protein induced suppression of adenylate cyclase and modulating of cAMP levels, hence regulating cellular metabolic functions (Colucci et al., 2023). Furthermore, GPR81 activation can cause more complex signaling networks, such as the PI3K-Akt/ERK pathways, causing the modulation of gene expression programs (Matsuhashi et al., 2025). A core manifestation of this signaling function is the promotion of neuroplasticity. Exercise-associated increases in circulating lactate have been linked to higher BDNF signaling (Müller et al., 2020), but the extent to which this effect is directly mediated by lactate–GPR81 signaling, rather than by other exercise-responsive mediators, remains to be clarified (Colucci et al., 2023).

Overall, the dual role of lactate in the brain—as an immediate energy provider and a long-term adaptive signaling trigger—forms a unified framework of action. Through this framework, exercise-driven lactate pulses can not only promptly experience the energy demands of neurons, but also, by activating specific receptor-induced signaling pathways, persistently remodel the brain, improve neuroplasticity, and improve cognitive function. This transforms short-term metabolic challenges into long-term advantages for brain health.

## 3 The multidimensional protective mechanisms of lactate signaling

**Figure 2:**
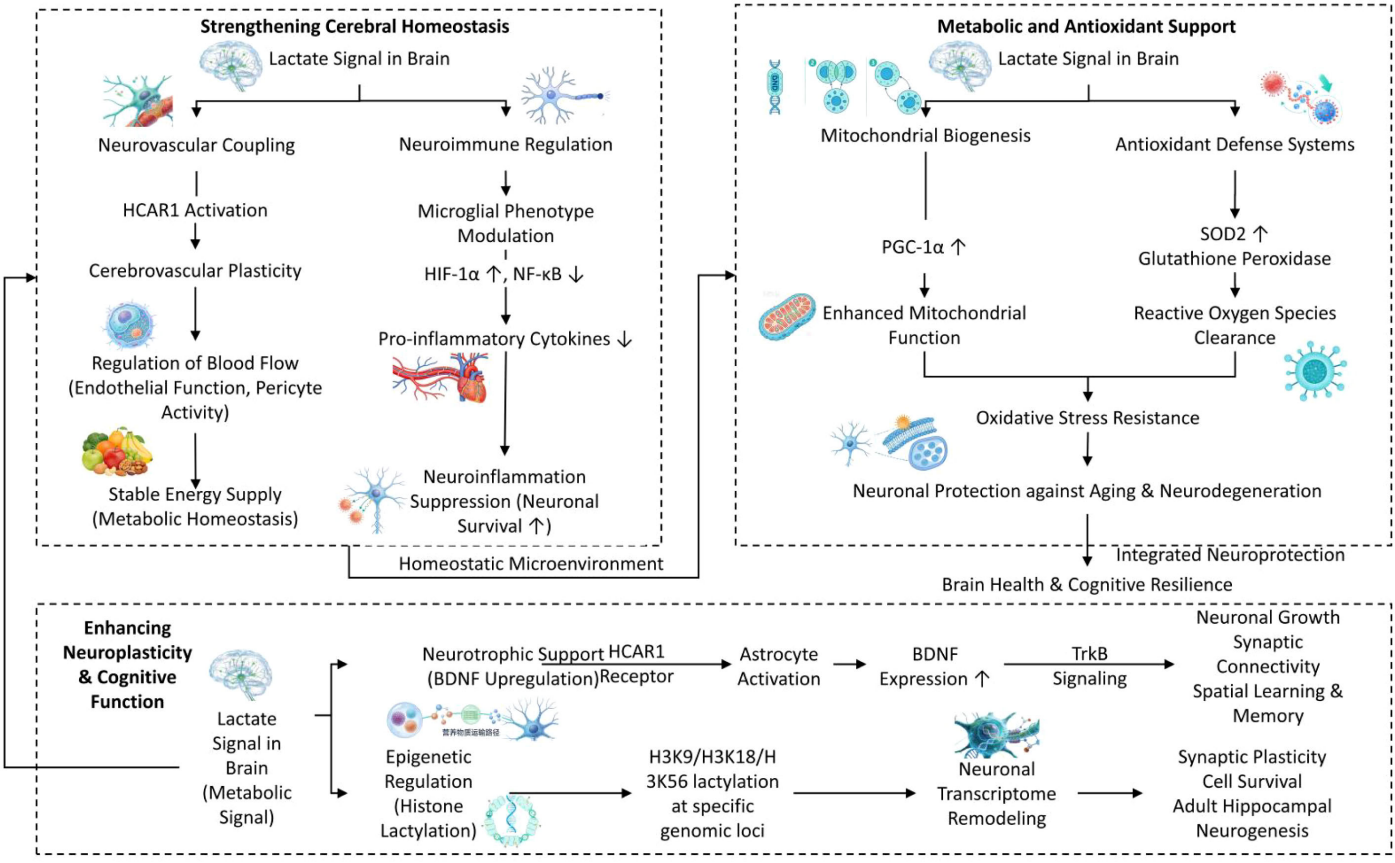
The multidimensional protective mechanisms of lactate signaling in the brain. This figure illustrates the major pathways through which lactate may support brain resilience. Lactate contributes to neuroplasticity through histone lactylation and neurotrophic signaling, including BDNF-related mechanisms. It may also help maintain cerebral homeostasis by influencing neurovascular coupling and modulating neuroimmune responses, particularly microglial inflammatory signaling. In addition, lactate may support mitochondrial function and antioxidant defense, thereby helping limit oxidative stress. These interconnected actions suggest that lactate is not only a metabolic substrate but also a pleiotropic signaling mediator linking peripheral exercise to adaptive responses in the brain. BDNF, brain-derived neurotrophic factor; HCAR1, hydroxycarboxylic acid receptor 1; HIF-1α, hypoxia-inducible factor 1α; NF-κB, nuclear factor kappa B; PGC-1α, peroxisome proliferator-activated receptor gamma coactivator 1α; SOD2, superoxide dismutase 2; TrkB, tropomyosin receptor kinase B.

Having established the journey of lactate from contracting muscle to the brain parenchyma, the focus now shifts to the question of functional consequence: once lactate reaches its central targets, what specific protective programs does it initiate? The answer lies in a multidimensional framework in which lactate operates not as a single-signal molecule but as a pleiotropic metabolic messenger capable of simultaneously engaging distinct yet interconnected protective pathways. These pathways span epigenetic reprogramming, neurotrophic support, hemodynamic regulation, immune modulation, and antioxidant defense—collectively forming an integrated network that safeguards neural structure and function. **Figure 2** illustrates the multidimensional protective mechanisms of lactate signaling in the brain.

### 3.1 Enhancing neuroplasticity and cognitive function

Lactate signaling is increasingly implicated in neuroplasticity and cognitive function. Exercise-driven lactate signaling can improve the remodeling of neural networks, increasing the brain’s adaptability to new environments and challenges.

#### 3.1.1 The emerging role of histone lactylation in epigenetic regulation

The function of lactate as a metabolic signaling molecule expands beyond its classic receptor-induced pathways into the key range of epigenetic regulation. Current evidence indicates that lactate can function as a novel substrate for histone modification, directly engaging in chromatin remodeling and gene transcription regulation, hence offering a new mechanism for determining how metabolic status instructs long-term changes in gene expression (Zhang et al., 2019). This discovery significantly improves the role of lactate as a central messenger connecting immediate cellular metabolism to long-term adaptive responses.

A key mechanism is that lactate-derived lactyl-CoA can function as a donor, facilitating the formation of a novel covalent modification—histone lactylation—on histone lysine residues (e.g., H3K9, H3K18, H3K56) (Zhang et al., 2019). This modification displays typical metabolic sensitivity: increases in intracellular lactate levels can dynamically and promptly drive an increase in histone lactylation. This means that lactate pulses driven by physiological activities, such as exercise can be directly translated into metabolic memory imprints on chromatin (Hu et al., 2025). The physiological significance of this mechanism is notably prominent in the nervous system. Evidence determines that lactate-induced histone lactylation is a core epigenetic switch modulating neuroplasticity and function. For example, lactate, by increasing histone H3K9 lactylation at specific genomic loci (e.g., genes linked to synaptic plasticity and cell survival), directly transforms the neuronal transcriptome, hence increasing the adaptability of neurons to environmental challenges and cognitive demands (Zhang YM. et al., 2025). notably significant is the vital role this pathway plays in adult hippocampal neurogenesis. Recent *in vivo* evidence shows that the brain’s lactate shuttle process directly modulates histone lactylation levels within the hippocampal dentate gyrus neural stem cell niche. This metabolism-dependent epigenetic modification, in turn, promotes the expression of genes linked to cell cycle and differentiation, regulating neural stem cell proliferation and the production of new neurons (Li Z. et al., 2025). This finding links the metabolic signal (lactate) generated by exercise with structural brain plasticity (neurogenesis) at the molecular level.

Hence, histone lactylation represents an emerging epigenetic language that links metabolic fluctuations (lactate levels) with persistent reprogramming of gene programs (neuroplasticity, neurogenesis). It explores how repeated exercise can cumulatively improve brain adaptability and resilience by writing on chromatin via repeated lactate signals, offering an unprecedented epigenetic explanation for exercise-induced brain benefits.

#### 3.1.2 Upregulation of neurotrophic support through BDNF and neurogenesis

BDNF is a central molecule modulating neurogenesis, synaptic plasticity, and neuronal survival. The ability of lactate, as a core metabolic messenger, to upregulate BDNF expression represents a core mechanism via which it facilitates brain health. This mechanism is induced, in part, via its specific receptor, HCAR1 (Müller et al., 2020; Pan et al., 2025). Evidence displays that in astrocytes, the binding of L-lactate to HCAR1 can activate downstream cellular signaling pathways, directly inducing BDNF expression (Foreman et al., 2025). These findings support the possibility that lactate contributes to neurotrophic signaling, although the relative importance of receptor-dependent and receptor-independent pathways remains unresolved.

Functionally, lactate signaling tightly interacts with the classic BDNF/tropomyosin receptor kinase B (TrkB) signaling pathway, jointly modulating neural network physiology (El Hayek et al., 2019; Griego and Galván, 2023). In hippocampal neurons, lactate/HCAR1 signaling has been displayed to regulate synaptic plasticity and synergize with BDNF/TrkB signaling to promote the physiological function of the CA3 network (Griego and Galván, 2023). This interaction directly links metabolic state to changes in synaptic efficacy, offering a mechanistic basis for determining the energy dependence of cognitive mechanisms. At the overall physiological level, exercise-driven lactate is a core messenger inducing the cognitive advantages of exercise. Evidence has uncovered that lactate discharged from muscles during exercise can cross the BBB and drive hippocampal Bdnf gene expression and TRKB signaling pathway activation via a SIRT1-dependent pathway. This lactate-dependent elevation in BDNF levels is directly linked to promoted spatial learning and memory retention in experimental animals (El Hayek et al., 2019). Hence, by upregulating BDNF, lactate not only facilitates the growth and differentiation of new neurons, but also increases the repair, remodeling, and functional connectivity of recent neural networks, finally promoting cognitive function and overall neural health. Overall, lactate and BDNF together constitute a core mediator circuit for exercise-driven neuroplasticity (Müller et al., 2020).

### 3.2 Strengthening cerebral homeostasis

Beyond its roles in energy support and plasticity-related signaling, lactate may also contribute to the maintenance of cerebral homeostasis, determining that the nervous system sustains functional stability and positively responds to challenges, such as metabolic fluctuations driven by exercise. This role is mainly reflected in the precise modulating of the neurovascular system and the active shaping of the neuroimmune environment.

The maintenance of brain function depends heavily on stable energy supply, determined by the tight coupling between neural activity, local metabolism, and cerebral blood flow, referred to as neurovascular coupling (Wang et al., 2025). Lactate plays an active signaling role in this mechanism (Lauritzen et al., 2014; Xue et al., 2022). Exercise not only enhances peripheral and central lactate levels, but also activates the lactate receptor HCAR1, impacting brain plasticity processes, comprising cerebrovascular plasticity (Xue et al., 2022). Lactate is increasingly considered one contributor to exercise-related neurovascular adaptation, acting within a broader network of systemic and local mediators (Huang et al., 2021). It may help regulate local cerebral blood flow by modulating vascular endothelial function, smooth muscle tone, or pericyte activity, hence determining adequate oxygen and nutrient supply to neurons and glial cells during periods of high metabolic demand (e.g., during cognitive tasks or post-exercise recovery) (Huang et al., 2021). This mechanism is critical for sustaining cerebral metabolic homeostasis and supporting sustained cognitive function.

In addition to strengthening energy delivery, lactate actively sustains neuroimmune homeostasis by regulating the function of the brain’s innate immune cells—microglia. Chronic or excessive neuroinflammation is a core factor causing neuronal damage and cognitive decline. Lactate illustrates a clear protective regulatory role in this perspective. In models of ischemic/hypoxic (oxygen-glucose deprivation) stress, lactate treatment can improve a modification in microglia toward a more protective phenotype (Liu and Zhou, 2024; Müller and Di Benedetto, 2025). This process involves activation of hypoxia-inducible factor-1α (HIF-1α) and suppression of the pro-inflammatory nuclear factor-kappa B (NF-κB) signaling pathway, hence significantly decreasing the generation of pro-inflammatory cytokines. This lactate-driven phenotypic shift in microglia ultimately enhances the survival rate of neurons in co-culture systems (Zhang et al., 2023). Hence, lactate is not only a metabolite in the astrocyte-neuron energy shuttle, but also a core signaling molecule capable of impacting microglial function and suppressing their induced neuroinflammation. Under physiological conditions and in the early stages of brain diseases, lactate exerts extensive neuroprotective effects by providing energy, transmitting signals, and supporting neurogenesis, and suppressing inflammation, making it a potential target for intervening in brain disorders (Liu and Zhou, 2024). In addition to astrocytes and microglia, endothelial cells and pericyte-associated vascular units are likely to shape how lactate influences local blood flow, barrier function, and inflammatory signaling in the aging brain (Lee et al., 2022).

Overall, lactate optimizes cerebral homeostasis via a dual pathway: on one hand, functioning as a vasoactive signal to modulate blood flow to experience metabolic demands; on the other, acting as an immunomodulatory signal to suppress harmful neuroinflammation. These two processes together offer a stable and supportive microenvironment for the brain when experiencing internal and external challenges, consolidating its status as a core metabolic messenger for sustaining brain health.

### 3.3 Metabolic and antioxidant support

The role of lactate in sustaining brain homeostasis expands beyond direct signal transduction and energy supply to include critical antioxidant defense systems, offering significant metabolic support for neural cells against oxidative stress—a key pathological factor in aging and NDs (Li et al., 2023; Liu and Zhou, 2024). It increases neuronal resistance to oxidative damage by improving the activity of endogenous antioxidant systems (Tauffenberger et al., 2019; Akter et al., 2023).

Research determines that this supportive effect has a clear molecular basis. For instance, direct administration of exogenous L-lactate into the rat hippocampus profoundly upregulates the expression of several core regulatory proteins linked to mitochondrial biogenesis (e.g., PGC-1α) and antioxidant defense (e.g., SOD2, glutathione peroxidase) (Tauffenberger et al., 2019). This finding reveals that lactate can increase mitochondrial function (the main source and clearance site of reactive oxygen species) and improve cellular antioxidant enzyme reserves by activating relevant metabolic and transcriptional programs. The mechanism of lactate’s antioxidant action is multi-layered, expanding beyond its role as a mere energy precursor. In addition to modulating gene expression via pathways possibly involving receptor signaling (e.g., HCAR1) or metabolic sensors (e.g., AMPK) as mentioned above, its derivative lactylation modification itself may directly engage in transcriptional regulatory networks, impacting gene programs linked to oxidative stress response and cell survival (Cao et al., 2025). Taken together, these findings suggest that lactate may support antioxidant defense through coordinated metabolic, transcriptional, and possibly epigenetic mechanisms.

Overall, The antioxidant role of lactate is a critical component of its integrative neuroprotective effects. By increasing mitochondrial health and improving antioxidant defenses, lactate may help limit accumulated oxidative damage because of metabolic stress or the aging process, hence offering a potential cellular metabolic basis for delaying cognitive decline and combating the development of NDs.

## 4 The failure of the lactate signaling axis in aging

**Figure 3:**
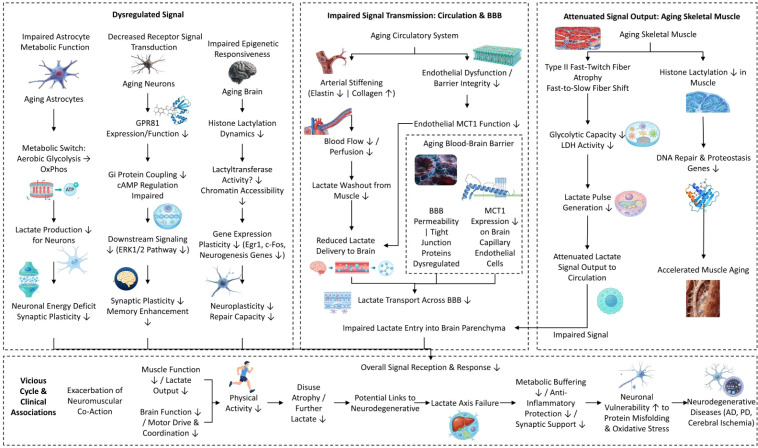
The failure of the lactate signaling axis in aging: from attenuated output to pathological consequences. This figure summarizes the proposed age-related disruption of the lactate signaling axis across three levels: signal generation, signal transmission, and signal response. In aged skeletal muscle, reduced glycolytic capacity may weaken exercise-encoded lactate output. In the circulation and at the blood-brain barrier, vascular dysfunction and impaired transporter activity may reduce lactate delivery to the brain. Within neural tissue, astrocytic metabolic changes, reduced receptor signaling, and impaired epigenetic responsiveness may further limit adaptive lactate actions. Together, these alterations may contribute to neuromuscular co-aging and increase vulnerability to neurodegenerative processes. AD, Alzheimer’s disease; BBB, blood-brain barrier; ERK1/2, extracellular signal-regulated kinase 1/2; GPR81, G protein-coupled receptor 81; LDH, lactate dehydrogenase; MCT1, monocarboxylate transporter 1; PD, Parkinson’s disease.

With increasing age, the function of the lactate signaling axis gradually declines, impacting the metabolic balance of muscles, the circulatory system, and the brain. Numerous physiological changes during the aging process interfere with the transmission and response of lactate signals, hence negatively influencing physical health and brain function. **Figure 3**. The failure of the lactate signaling axis in aging: from attenuated output to pathological consequences.

### 4.1 Attenuated signal output: skeletal muscle aging and declined exercise capacity

Skeletal muscle is not only an organ of movement, but also the main source of lactate, a core signaling molecule generated during exercise. The significant structural degeneration and functional decline of skeletal muscle during aging directly cause a significant attenuation of its capacity as a lactate signal generator, representing the initial step in the dysfunction of the lactate shuttle system (Luo et al., 2025).

The progressive loss of skeletal muscle mass and strength triggered by aging is central to the frailty syndrome. Muscle aging is tightly related to neurological decline, and the characteristic severe muscle atrophy significantly impacts the quality of life in the elderly. Proteomic evidence displays that the molecular and cellular changes in skeletal muscle during aging comprise extensive proteomic modifications and shifts in muscle fiber type (Dowling et al., 2023). It is critical to acknowledge that during aging, fast-twitch fibers (type II) are more prone to atrophy than slow-twitch fibers, causing a tendency for a fast-to-slow fiber shift in skeletal muscle (Dowling et al., 2023). Furthermore, fast-twitch fibers count on the glycolytic pathway for energy supply, possess high lactate dehydrogenase (LDH) activity, and generate large amounts of lactate during high-intensity, anaerobic exercise (Smith and Plowman, 2007). From a metabolic function perspective, aging differentially impacts the metabolic adaptation of various fiber types. Single-fiber proteomic analysis reveals that although mitochondrial content declines with age in both fast and slow fibers, glycolytic and glycogen metabolic pathways are upregulated in slow fibers, but downregulated in fast fibers (Murgia et al., 2017). This implies that the glycolytic capacity of the fast-contracting units responsible for producing high lactate pulses is inhibited during aging, directly restricting the efficient synthesis of lactate. This metabolic decline is also implied at the epigenetic level. Histone lactylation, a modification effectively linked to glycolytic activity, has been uncovered to counteract cellular senescence and skeletal muscle aging (Meng et al., 2025; Sun et al., 2025). During aging, glycolysis is inhibited within skeletal muscle, and histone lactylation levels then decrease. This decrease in lactylation can downregulate the expression of key genes in aging-associated pathways, such as DNA repair and proteostasis, accelerating the muscle aging process. Exercise can counteract this mechanism by enhancing histone lactylation (Meng et al., 2025).

The integrated consequence is that aged muscle has a decreased capacity to generate lactate pulses of sufficient amplitude in response to exercise demands. Since lactate is not only an energy metabolite, but also a core signaling molecule improving exercise-driven adaptive remodeling (including benefits for the brain) (Nalbandian and Takeda, 2016), its decreased generation directly impairs the body’s adaptability to high-intensity exercise. This manifests as reduced exercise endurance, enhanced muscle fatigue, and significant recovery time, as imbalances in lactate generation and clearance directly impact fatigue monitoring and recovery (Huang et al., 2025). More importantly, this significantly impairs lactate’s regulatory effect on the entire organism (notably the brain) as a messenger molecule capable of remodeling brain function and enhancing cognition (Zhu et al., 2025).

Thus, skeletal muscle aging may reduce the capacity to generate exercise-encoded lactate pulses, potentially weakening one upstream component of the broader lactate signaling framework during aging.

### 4.2 Impaired signal transmission: dysfunction of the circulatory system and BBB

Even if lactate is generated in muscle, its effective transport within the aging organism experiences numerous obstacles (Wang MY. et al., 2024; Mayorga-Weber et al., 2025). From the circulation to the blood–brain barrier, several components of lactate transport may become less efficient with age, thereby reducing the reliability of lactate transfer as an inter-organ metabolic signal (Wang MY. et al., 2024; Mayorga-Weber et al., 2025).

Aging is associated with a functional decline of the circulatory system, directly impacting the kinetics of lactate entry from muscle tissue into the systemic circulation and its arrival at distal organs (Zhang et al., 2025). Structural changes in arterial walls, such as elastin decrease and collagen increase, cause arterial stiffening, decreasing vascular distensibility and compliance to pulsatile flow, hence altering hemodynamics (Xuereb et al., 2023). Blood flow is a core factor impacting lactate clearance from muscle, and under impaired hemodynamic conditions, decreased blood flow or perfusion delays the process of lactate being washed into the systemic circulation (Sañudo et al., 2020). Furthermore, vascular aging is linked to endothelial dysfunction, presented as impaired endothelial barrier function and a decreased capacity to maintain appropriate permeability and metabolite transport. These changes jointly contribute to altered physiological homeostasis in the elderly (Li Q. et al., 2025). Lactate transport across endothelial cells through specific MCTs (MCTs), such as MCT1, plays a critical role in systemic metabolism and interorgan fuel partitioning; Hence, the transport capacity of endothelial cells directly influences blood lactate concentration changes (Zhang et al., 2021).

When lactate reaches the brain through the bloodstream, the age-associated dysfunction of a second critical barrier—the BBB —turns into a more significant limiting factor. Evidence displays that in physiological aging, BBB function is impaired with enhanced permeability, tightly linked to changes in the senescent phenotype of cerebrovascular endothelial cells and imbalances in the regulation of tight junction proteins (Novo et al., 2024). Aging causes enhanced BBB permeability associated with dysregulated expression of tight junction proteins, triggering enhanced inter-endothelial gaps and compromised barrier integrity (Andjelkovic et al., 2023).

One likely consequence of these vascular and transporter-level alterations is reduced efficiency of lactate exchange across the blood–brain barrier. It is more critical that the expression and function of the key transmembrane transport proteins for lactate—MCTs (MCTs, notably MCT1)—may also be impaired in the aging brain (Pérez-Escuredo et al., 2016; Mayorga-Weber et al., 2025). Evidence on the corpus callosum found lactate accumulation, decreased glucose levels, and oxidative stress in the corpus callosum of aged mice. Further evidence suggested that aged axons exhibit a decreased ability to maintain oxidative energy metabolism during electrical stimulation, while the mechanisms of glycolysis, lactate dehydrogenase-induced lactate generation, and MCT-induced lactate transport—all relied upon by young axons—are significantly impaired in aged axons (Mayorga-Weber et al., 2025). Concurrently, observations of decreased lactate uptake and decreased MCT1 expression during aging similarly disrupt lactate flux and oxidation, causing inefficient energy utilization, which may trigger oxidative stress and axonal degeneration (Mayorga-Weber et al., 2025). MCT1 is a core transporter for lactate release from astrocytes and the entry of exogenous lactate (e.g., from exercise) across the BBB into the brain (Liu and Zhou, 2024).

The impediment of lactate signal transmission has significant implications. As the end-product of glycolysis, lactate is not only an energy substrate and metabolite, but also a signaling molecule for the novel epigenetic modification of protein lactylation, critical for energy metabolism and signal transduction in brain tissue under physiological and pathological conditions (Li et al., 2023). Hence, age-associated barriers to lactate entry into the brain not only impact cerebral energy homeostasis, but may also impair lactate’s regulatory effects on neurogenesis, neuroplasticity, endothelial function, and microglial function—all vital for emotional and cognitive health (Zhang S. et al., 2025).

Overall, the dysfunction of the circulatory system and the BBB jointly represents a relay attenuation in the transmission link of the lactate signaling axis, making it difficult for protective signals from muscles to positively reach and act upon the aging brain.

### 4.3 Dysregulated signal reception and response

Aging also impacts the reception and response to lactate signals. Receptors and cellular functions involved in lactate signal transduction in the brain gradually deteriorate with age, causing decreased effectiveness of lactate signal transduction.

#### 4.3.1 Impaired metabolic function of astrocytes

Astrocytes are central regulators of brain metabolic homeostasis, being vital in the generation and distribution of lactate. The classic astrocyte-neuron lactate shuttle hypothesis offers the foundational framework for determining this process. This hypothesis posits that lactate discharged by astrocytes into the extracellular space can be taken up and metabolized by neurons; thus, astrocyte-derived lactate is a critical energy substrate for neurons (Kim et al., 2025). Furthermore, current evidence further explains that lactate is not only an energy source, but also a core metabolic signaling molecule impacting significant functions, such as synaptic plasticity (Wu et al., 2023).

Nevertheless, during aging, this key metabolic function of astrocytes is significantly inhibited. Evidence reveals that astrocytes in the aging brain experience a critical metabolic switch: their energy metabolism shifts from aerobic glycolysis (which effectively generates lactate) towards oxidative phosphorylation. This intrinsic metabolic reprogramming directly causes a reduced ability to supply lactate to neurons (Acevedo et al., 2023). This shift associates with the inherent energy deficit generally uncovered in aging neural tissue. The consequences are twofold: on one hand, as an energy substrate, decreased lactate supply may trigger neuronal energy hunger; on the other hand, as a signaling molecule, decreased lactate flux impairs its ability to modulate synaptic plasticity and neuroprotective gene expression (Wu et al., 2023).

Hence, this age-associated impairment of astrocyte metabolic function directly disrupts the effectiveness of the brain’s internal lactate metabolic microcirculation. It impacts not only immediate energy supply, but also impairs the long-term adaptive signal transmission induced by lactate, which is regarded as a significant cellular metabolic basis for cognitive decline in the elderly (Acevedo et al., 2023).

#### 4.3.2 Decreased effectiveness of receptor signal transduction

The key to lactate’s signaling function in the brain lies in its binding to its specific receptor—GPR81. This receptor is functionally expressed not only in adipose tissue, but also in the mammalian brain, inducing lactate signaling to neurons (Morland et al., 2015). Thus, lactate is not just an energy substrate, but also an endogenous ligand for GPR81, playing a signaling role in the CNS (Colucci et al., 2023).

However, with advancing age, the effectiveness of this key signal-receiving apparatus may significantly decline. The function of GPR81 resembles a metabolic sensor, coupling energy metabolism, synaptic activity, and blood flow regulation (Morland et al., 2015; Colucci et al., 2023). Its activation, through Gi proteins, downregulates adenylate cyclase, reduces intracellular cAMP levels, and hence modulates numerous downstream pathways. Under pathological conditions (e.g., cerebral ischemia), activation of GPR81 displays neuroprotective potential, determining the integrity of its signaling pathway is critical for brain health (Colucci et al., 2023). Nevertheless, during aging, the expression level, membrane localization, or coupling effectiveness with downstream signaling proteins of the receptor itself may decrease, causing decreased signal transduction effectiveness (de Oliveira et al., 2019; Martín and Dotti, 2022).

This attenuation of signaling at the receptor level has direct functional consequences. The lactate/GPR81 signaling pathway has been indicated to play a core role in hippocampal synaptic plasticity and memory enhancement (Bian et al., 2026). For example, lactate driven by high-intensity interval training facilitates hippocampal synaptic remodeling and promotes memory through GPR81 signaling and its downstream ERK1/2 pathway (Bian et al., 2026). Hence, decreased effectiveness of GPR81 signaling implies that lactate’s regulatory effects on neuroplasticity, anti-inflammatory responses, and cognitive function are impaired. Ultimately, lactate’s dual role in the brain—as both an energy substrate and a signaling molecule involved in signal transduction and epigenetic modification (Wang MY. et al., 2024)—cannot be completely determined during aging, causing decreased brain adaptability, repair capacity, and overall cognitive function (Wu et al., 2023).

#### 4.3.3 Impaired epigenetic responsiveness

Lactate’s modulating of brain function happens not only via immediate signal transduction, but also via epigenetic processes that exert lasting effects on gene expression. In a young, metabolically active brain, lactate functions as a vital bridge connecting cellular metabolic state to gene transcription programs by facilitating a novel histone modification—histone lactylation (Zhang et al., 2019; Wang Y. et al., 2024). The lactate shuttle process controls histone lactylation levels, and the latter, as a core node, modulates key mechanisms, such as adult hippocampal neurogenesis, hence closely linking metabolic state to gene regulation in neural development (Li Z. et al., 2025). Under physiological conditions, this lactate-induced histone lactylation can finely tune the expression of genes linked to neuroplasticity (e.g., Egr1, c-Fos) in response to synaptic activity (Zhang YM. et al., 2025).

However, the aging mechanism is associated with a general rigidity of the epigenetic landscape and a decline in metabolic flexibility, which directly impairs lactate’s capacity as an epigenetic regulator (Chen et al., 2025; Sun et al., 2025). Although the precise molecular processes are still being investigated, it is foreseeable that in the aging brain, the dynamic effectiveness of histone lactylation modification and its regulatory capacity over downstream genes may decrease because of decreased fluctuations in lactate levels, modified activity or expression of enzymes involved in lactylation (e.g., lactyltransferases), or changes in chromatin accessibility (Datta and Chakrabarti, 2018; Zhang YM. et al., 2025). This impaired lactate-dependent epigenetic responsiveness implies the brain becomes less capable of positively altering its gene expression profile in response to metabolic signals, such as exercise-driven lactate pulses (Li et al., 2023; Zhang YM. et al., 2025). The direct consequence is that the brain’s ability to undergo adaptive changes and repair via epigenetic reprogramming in the face of external stimuli or damage is significantly compromised, hence triggering age-associated loss of neuroplasticity and cognitive decline (Zhang YM. et al., 2025).

### 4.4 Vicious cycle and clinical associations

#### 4.4.1 Exacerbation of neuromuscular co-aging

Impairment of lactate-related muscle–brain communication may represent one reinforcing mechanism within the bidirectional decline of muscle and brain function during aging. As skeletal muscle mass and function progressively decline, its glycolytic capacity and response to exercise demands weaken, directly causing attenuated production of the key signal, lactate (Shur et al., 2021; Meng et al., 2025). Evidence determines that the molecular framework of skeletal muscle aging involves a integrative decline in energy substrate handling capacity and alterations in the generation and utilization patterns of metabolites, such as lactate, jointly influencing exercise endurance and recovery (Horlem et al., 2025). concurrently, the effectiveness of the lactate shuttle metabolic pathway connecting muscle to brain decreases during aging, impairing lactate’s ability to induce exercise-driven cognitive enhancement as both an energy substrate and a signaling molecule (Xue et al., 2022).

Concurrently, the brain’s capacity to receive and respond to lactate signals also weakens. As a core metabolic and signaling mediator connecting peripheral metabolic changes to central synaptic function, plasticity, and cognitive performance, defects in the lactate signaling pathway directly weaken these adaptive responses (Han et al., 2025). The result is a decline in the brain’s repair capacity and neuroplasticity, potentially accelerating neurodegenerative processes, as alterations in lactate metabolism have been indicated to be linked to the development of NDs (Cao et al., 2025).

Taken together, these observations support a self-reinforcing model in which declining muscle function, reduced metabolic signaling capacity, impaired brain adaptation, and lower physical activity may amplify one another over time. Importantly, this model does not imply that reduced lactate signaling is the sole primary driver of neuromuscular co-aging, but rather that it may act as a biologically plausible mediator within a broader network of aging-related processes.

#### 4.4.2 Potential links to NDs

Age-related impairment of lactate signaling may intersect with several established pathogenic mechanisms in neurodegenerative diseases, such as AD and Parkinson’s disease (PD) (Liu and Zhou, 2024; Wang MY. et al., 2024). Evidence displays that alterations in lactate metabolism and its signaling roles are a general feature during the occurrence and development of NDs, and changes in its levels may impact the metabolic adaptability of neurons, hence accelerating disease development (Wang MY. et al., 2024). Lactate in the brain functions not only as an energy substrate, but also as a metabolic signaling molecule impacting synaptic activity and neurodegeneration, implying that interruption of lactate signaling impairs neuronal metabolic resilience under stress (Wu et al., 2023). This connection is built via lactate’s numerous protective roles in the brain. Lactate extensively engages in the pathophysiological mechanisms of brain diseases, such as AD, PD, and cerebral ischemia by suppressing microglia-induced neuroinflammation, offering energy, functioning as a signaling molecule, and imposing neuroprotective effects (Liu and Zhou, 2024). Moreover, the lactate-sensing receptor GPR81, expressed in the CNS, can couple energy metabolism, synaptic activity, and cerebral blood flow. Lactate signaling via GPR81 may play a role in counteracting disease damage by supporting neuroprotection and regulating cerebral blood flow (Colucci et al., 2023).

Within the aging context, impairment across the generation–transport–response continuum of lactate signaling may create a permissive environment for neurodegenerative progression rather than acting as a single disease-specific trigger. The failure of the lactate signaling axis deprives the brain of a significant system for metabolic buffering, anti-inflammatory protection, and synaptic support, making neurons more vulnerable when experiencing pathogenic factors, such as protein misfolding and oxidative stress. These observations support further investigation of lactate-related pathways as potential adjunctive targets for delaying or modifying neurodegenerative progression.

#### 4.4.3 Integrating lactate signaling with canonical neurodegenerative pathways

Impaired lactate handling may intersect with amyloid-β pathology by weakening neuron–glia metabolic coupling and reducing the resilience of the neurovascular unit. In Alzheimer’s disease, CSF lactate abnormalities have been reported across the disease continuum and appear to relate to blood–brain barrier integrity, suggesting that lactate dysregulation may emerge within a broader Aβ-linked metabolic-vascular milieu rather than as an isolated event (Liguori et al., 2015; Zebhauser et al., 2022; Liu and Zhou, 2024).

Tau-associated neurodegeneration appears to show a closer relationship to lactate disturbance than amyloid pathology alone. In AD, CSF Lactate has been linked to tau-related changes and cognitive decline, supporting the view that lactate dysregulation may track with tau-associated neuronal injury and energetic failure (Liguori et al., 2015). Mitochondrial dysfunction is a major point of convergence between canonical neurodegeneration and impaired lactate signaling. Elevated CSF lactate and its association with cerebral glucose hypometabolism support the possibility that disrupted lactate utilization reflects or amplifies mitochondrial stress in vulnerable brain networks (Liguori et al., 2015; Liguori et al., 2016). Lactate-related pathways also intersect with neuroinflammation, particularly through microglial state regulation. Experimental evidence indicates that lactate can influence microglial inflammatory signaling, while aberrant microglial lactylation has been implicated in brain aging and AD-related inflammatory amplification (Wei et al., 2023; Liu and Zhou, 2024). Oxidative stress represents another likely bridge between lactate dysregulation and neurodegeneration. When lactate transport or utilization becomes inefficient, the resulting metabolic mismatch may reduce antioxidant resilience and increase oxidative burden, thereby exacerbating neuronal vulnerability in aging and disease (Liguori et al., 2016; Wei et al., 2023).

## 5 The intervention prospects of targeting the lactate system

As the significance of lactate in aging and neuromuscular degeneration obtains increasing attention, intervention approaches targeting the lactate signaling system are commonly proven to be potential therapeutic avenues. These interventions seek not only to promote lactate generation and transport, but also to promote its role within the brain, hence increasing neuroplasticity, improving cognitive function, and slowing age-associated disease development. **Table 1** outlines intervention prospects for targeting the lactate system in aging and neurodegeneration.

**Table 1:** Intervention prospects for targeting the lactate system in aging and neurodegeneration.

Strategy	Mechanistic rationale	Current evidence level	Key translational limitations and representative references
Individualized exercise prescription	Exercise is the most physiological way to generate transient lactate pulses and may simultaneously enhance lactate production, circulatory transport, and brain responsiveness. Appropriate control of intensity, duration, and training structure may help optimize lactate kinetics rather than simply maximize lactate accumulation.	Moderate preclinical/physiological human evidence; limited direct causal clinical evidence. Exercise–lactate relationships are well established physiologically, and animal studies support lactate-related neuroplastic and vascular effects. However, direct human evidence linking lactate-guided exercise prescriptions to neuroprotection remains limited.	Defining an “optimal” lactate profile remains difficult across ages and disease stages; inter-individual variability in metabolic fitness and lactate threshold is substantial; direct biomarkers confirming brain target engagement in humans are still limited. Representative refs (Beneke et al., 2011; Nalbandian and Takeda, 2016; Casado et al., 2023; Zhu et al., 2025):;
GPR81/HCAR1-targeted pharmacology	Pharmacological activation of GPR81/HCAR1 may mimic part of lactate signaling when endogenous production, transport, or responsiveness is impaired. This strategy is conceptually attractive for patients unable to exercise adequately.	Predominantly preclinical evidence. Experimental studies suggest potential effects on neuroplasticity, vascular signaling, and injury responses, but robust clinical neurodegeneration data are lacking.	[1]Receptor biology is strongly context-dependent; effects may differ between acute injury and chronic neurodegeneration; optimal dose, timing, and tissue specificity remain unclear; no established clinical efficacy in aging-related NDs. Representative refs (Hu et al., 2020; Colucci et al., 2023):.
Ketogenic or ketone-based metabolic interventions	Ketogenic strategies may reshape systemic and cerebral energy metabolism, provide alternative oxidative substrates, and influence signaling pathways relevant to oxidative stress and neuronal resilience. These interventions may indirectly modify the lactate-related metabolic environment.	Early clinical and translational evidence, but heterogeneous. Small studies and reviews suggest possible benefits in MCI, AD, and PD, but findings remain inconsistent and population-specific.	Adherence burden is high; dietary restrictiveness and metabolic side effects may limit long-term use; patient tolerance varies; long-term efficacy and safety in older adults with neurodegenerative disease remain uncertain. Representative refs (Lilamand et al., 2022; Bohnen et al., 2023):
Lactate or precursor supplementation	Exogenous lactate or its precursors may directly increase circulating substrate availability and potentially engage receptor-mediated and lactylation-related pathways. This is the most direct method for manipulating lactate exposure.	Preclinical evidence with limited early human data. Mechanistic plausibility is high, and emerging human studies suggest short-term metabolic effects, but durable clinical benefit is unproven.	[2]The therapeutic window may be narrow; tissue distribution, clearance, and compartment-specific effects are difficult to control; chronic accumulation or impaired clearance may become maladaptive rather than beneficial. Representative refs (Fang et al., 2024; Wang MY. et al., 2024; Kemna et al., 2025):
Biomarker and imaging framework	Lactate-related biomarkers may help quantify dysfunction across the production–transport–response continuum of the lactate signaling axis. Candidate measures include CSF lactate, transporter expression, receptor-related signaling indices, histone lactylation markers, and MRS-based estimates of lactate flux.	Promising but exploratory. CSF lactate and imaging-based markers have translational relevance, but their specificity and clinical interpretability remain limited.	[3]Standardization across sample type, measurement timing, and analytic methods is lacking; systemic metabolic confounding remains substantial; longitudinal validation for routine clinical use is still insufficient. Representative refs (Liguori et al., 2015; Kirov and Tal, 2020; Li et al., 2023):

### 5.1 Precision behavioral interventions to promote exercise-driven lactate signaling

A central goal of precision behavioral interventions is to design individualized exercise prescriptions that appropriately engage lactate dynamics without assuming that larger lactate elevations are always better (Beneke et al., 2011; Casado et al., 2023). Exercise intensity, mode, and individual training status all significantly impact the kinetics of lactate generation and its ensuing physiological effects.

The individual’s metabolic response to exercise load forms the basis for precision intervention. Evidence indicates that at exercise intensities below the assessed lactate threshold, the kinetics of ventilation and carbon dioxide output are closely coupled, implying a stable metabolic modulating pattern during submaximal exercise (Ward et al., 2023). For chronically sedentary individuals, even during low-intensity exercise, their active skeletal muscles mainly count on carbohydrates for fuel, a metabolic inflexibility potentially associated with lower mitochondrial content (Barakati et al., 2023). These individual differences suggest that a uniform exercise intensity may generate vastly various lactate signals; Hence, interventions require to be individualized. Moreover, training cycles and the distribution of exercise intensity are core variables for regulating lactate signals. Resting blood lactate levels in athletes fluctuate with training cycles (e.g., pre-season, in-season, off-season) and link to metabolic capacity during high-intensity exercise. Lower resting lactate and higher post-exercise lactate clearance rates during the season determine better metabolic adaptation (Kano and Sato, 2022). In endurance athletes, an 8-week study comparing various training intensity distributions (e.g., pyramidal vs. polarized) integrated with plyometric training displayed that both models positively promoted physiological metrics linked to lactate metabolism (e.g., running speed at 4 mmol/L blood lactate concentration) and 5-km time trial performance, with added advantages from plyometric training (Filipas et al., 2023). This illustrates that empirically integrating various training intensities can exactly map an individual’s lactate threshold and clearance capacity. It is more significant that the molecular processes via which exercise benefits the brain are induced by lactate, offering a target for precision interventions. Animal experiments determine that exercise can improve cerebral angiogenesis by activating the lactate receptor HCAR1 (GPR81), hence triggering the ERK1/2-PI3K/Akt signaling pathway (Zhang et al., 2022). These observations support the idea that exercise prescriptions should aim to optimize, rather than simply maximize, lactate responses in accordance with age, training status, and disease context.

Hence, precision behavioral interventions seek to move beyond one-size-fits-all exercise advice. By exploring an individual’s metabolic profile, training status, and recovery capacity, it tailors exercise intensity, duration, and mode. The goal is to positively enhance lactate generation within safe limits to offer sufficient signaling substrate; concurrently, to promote circulatory function and lactate clearance effectiveness, determining the signal is positively transported to the brain; and via regular, periodic stimulation, to promote the brain’s receptor response and downstream pathway adaptation to the lactate signal. This method, which fails to count on pharmaceuticals, provides high safety and sustainability, offering a powerful non-pharmacological strategy for strengthening endogenous lactate signaling and sustaining brain health in individuals with various physiological statuses, comprising healthy aging and early stages of NDs. At present, support for exercise-based modulation of lactate signaling is strongest at the physiological and preclinical levels, whereas direct causal human evidence linking lactate-targeted exercise prescriptions to neuroprotection remains limited (Nalbandian and Takeda, 2016; Zhu et al., 2025).

### 5.2 Pharmacological strategies: GPR81 agonists and metabolic modulators

Pharmacological interventions provide a direct and powerful tool to handle the dysfunction of the lactate signaling axis during aging. The key strategy involves progressing receptor agonists to directly mimic or increase lactate signaling, or utilizing metabolic modulators to promote endogenous lactate generation and metabolic cycling. Given that the GPR81 receptor is the core molecule via which lactate exerts its signaling function in the CNS, and its expression or function may decline with age, it represents a rational target for drug intervention. The development of selective GPR81 agonists has been proposed as a strategy to bypass limitations in endogenous lactate availability or signaling efficiency, but this approach remains complicated by context-dependent receptor biology. Preclinical evidence offers proof-of-principle. For example, in a rat model of traumatic brain injury, L-lactate pretreatment, by increasing GPR81 signaling, significantly upregulated the expression of proteins linked to synaptic plasticity (e.g., PSD95, GAP43, BDNF) in the cortex and hippocampus and delayed neurological deficits (Zhai et al., 2020). This reveals that pharmacologically activating GPR81 holds promise for mimicking exercise-driven lactate signals, hence promoting neuroplasticity, neurotrophic support, and functional recovery. It is worth noting that the role of lactate/GPR81 signaling is context-dependent. In cerebral ischemia models, evidnece uncovered that suppressing GPR81 decreased neuronal death, revealing lactate might trigger ischemic injury through GPR81 activation (Shen et al., 2015). These apparently divergent findings suggest that the biological consequences of GPR81 activation are likely shaped by timing, anatomical location, disease state, and the balance between adaptive and maladaptive signaling. An ideal agonist may require spatiotemporal specificity or conditional activation properties to prevent possible risks in acute severe injury while imposing protective effects in chronic aging or degenerative nechanisms. In the neurodegeneration field, evidence for pharmacological targeting of GPR81/HCAR1 remains overwhelmingly preclinical, and its clinical translatability is still uncertain because receptor effects appear to vary with tissue context and disease stage (Shen et al., 2015; Hu et al., 2020; Colucci et al., 2023).

### 5.3 Nutritional and metabolic interventions: ketogenic diet and precursor supplementation

Beyond behavioral and pharmacological strategies, nutritional interventions targeting metabolic pathways provide another prospective avenue for regulating the lactate system. These interventions mainly revolve around two approaches: one uses the ketogenic diet to indirectly impact the energy metabolism landscape; the other involves directly complementing lactate or its metabolic precursors.

The ketogenic diet, as an extreme metabolic intervention, compels the liver to convert fatty acids into ketone bodies (e.g., β-hydroxybutyrate) by drastically decreasing carbohydrate intake and enhancing fat proportion. Under physiological conditions, the brain mainly utilizes glucose for energy, but during glucose scarcity (e.g., significant fasting), ketone bodies become a significant alternative fuel (Jensen et al., 2020). Its importance expands beyond offering an alternative fuel. Evidence determines that ketone bodies have complex biological effects beyond energy metabolism, comprising regulating neuronal excitability, impacting gene expression in response to oxidative stress, and acting as signaling molecules within brain cells (García-Rodríguez and Giménez-Cassina, 2021). At the clinical level, ketogenic interventions have been explored to handle the energy crisis in NDs, such as AD, which are defined by weakeded brain glucose metabolism (Jensen et al., 2020). It is significant to imply that the influence of a ketogenic diet on systemic energy substrate metabolism is complex and needs individual consideration. For instance, in patients with pyruvate dehydrogenase deficiency, its utilization needs balancing glucose tolerance and lactate levels (Stenlid et al., 2014).

Lactate and its precursor supplementation denote a more direct strategy. Lactate has been redefined from a glycolytic waste product to an energy-offering substrate and a signaling molecule regulating cellular function, with its derived lactylation modification playing roles in the pathology of different diseases (Liu et al., 2024). Hence, directly complementing exogenous lactate or its direct precursors (e.g., sodium pyruvate) seeks to enhance lactate levels in the circulation and target tissues. This provides dual underlying benefits: on one hand, offering an easily utilizable alternative oxidative substrate for the aging brain with weakened energy metabolism; on the other hand, offering sufficient ligand concentration for its signaling functions (e.g., through GPR81 receptor or histone lactylation). Although excessive lactate accumulation is linked to different disease states, it also plays beneficial roles in modulating immune responses, increasing exercise performance, and neural signaling (Kumar et al., 2025). Hence, precise regulation of lactate metabolism has turned into a prospective therapeutic direction (Liu et al., 2024). For nutritional and metabolic interventions, early human studies and clinical observations are available—particularly for ketogenic strategies and, more recently, lactate infusion—but the overall evidence base remains limited and heterogeneous, and long-term efficacy and safety in aging-related neurodegeneration remain to be established (Lilamand et al., 2022; Bohnen et al., 2023; Kemna et al., 2025).

### 5.4 Biomarker development: from diagnosis to effectiveness monitoring

As the role of lactate in aging and NDs becomes clearer, progressing biomarkers capable of quantifying the function of the lactate signaling system has turned into a core and extremely translational evidence perspective. These biomarkers seek to evaluate the integrity of the lactate signaling axis, offering an objective basis for early diagnosis, risk stratification, disease development monitoring, and effectiveness assessment of interventions (Jin et al., 2024). Potential biomarker candidates extend beyond lactate concentration alone and may include transporter expression, receptor-related signaling activity, histone lactylation status, and imaging-based estimates of lactate flux (Kirov and Tal, 2020; Li et al., 2023).

Detecting lactate concentrations in body fluids, such as blood and cerebrospinal fluid (CSF) offers critical information about systemic and central energy metabolic status (Belu et al., 2025). In pathological states, such modifications are notably significant. For example, in AD patients, CSF lactate levels are significantly increased, with concentrations higher in mild AD patients than in moderate-to-severe ones. Further evidence uncovered that higher CSF tau protein levels were poorly inked to lower lactate concentrations, revealing that tau protein toxicity may concurrently weaken mitochondrial function and glycolysis. This dynamic change in lactate metabolism is tightly associated with cognitive decline (Liguori et al., 2015). These observations suggest that altered lactate profiles may reflect disease-related metabolic disturbance, but their specificity is limited unless interpreted alongside complementary molecular, cellular, or imaging markers. For biomarker development, current support is promising but still exploratory: CSF lactate, lactylation-related markers, and MRS-based readouts have translational potential, yet specificity, standardization, and longitudinal validation remain insufficient for routine clinical use (Liguori et al., 2015; Kirov and Tal, 2020; Li et al., 2023).

### 5.5 Future challenges and key issues

Despite increasing interest in targeting lactate-related pathways, major conceptual and translational challenges remain. A major unresolved issue is that lactate may be adaptive when generated as a transient, exercise-encoded signal but maladaptive when chronically accumulated, poorly cleared, or compartmentalized within pathological tissue microenvironments (Fang et al., 2024). Abnormal lactate metabolism may reflect not only excessive production but also impaired transport, reduced cellular uptake, and defective clearance, all of which can distort local metabolic signaling (Wang MY. et al., 2024). When clearance is insufficient, lactate may accumulate within restricted tissue compartments rather than behaving as a transient exercise-encoded pulse, thereby shifting from adaptive signaling toward maladaptive metabolic persistence (Fang et al., 2024; Wang MY. et al., 2024). In the aging brain, transporter dysfunction and barrier-level abnormalities may further promote regional lactate retention and inefficient utilization, especially when MCT-dependent flux is compromised (Wang MY. et al., 2024; Mayorga-Weber et al., 2025). Under these conditions, a more acidosis-like microenvironment may emerge, with potential consequences for inflammatory tone, cellular stress responses, and tissue vulnerability. Therefore, future interventions should aim not simply to elevate lactate levels, but to restore appropriate lactate kinetics, spatial distribution, and clearance across tissues (Wang MY. et al., 2024).

How to exactly modulate the production and action of lactate without driving excessive metabolic burden or other detrimental effects is a critical issue. Moreover, individual variability in the lactate signaling axis diaplays a challenge for the extensive utilization of intervention approaches. Differences among individuals in lactate metabolism, lactate signal reception, and expression of associated receptors necessitate individualized intervention plans. Furthermore, the numerous physiological modifications during aging may cause various effects of lactate signaling. Determining the features of lactate signaling at various stages of aging and developing targeted treatment approaches are core issues for future evidence. The correlation between lactate and NDs is not yet completely determined. Although recent evidence reveals possible functions of lactate in these diseases, more clinical trials and fundamental evidence are required to further confirm the effectiveness and safety of targeting the lactate signaling axis in treatment.

## 6 Conclusion and outlook

This review integrates current evidence on the biological role of lactate in aging and proposes the lactate signaling axis as a useful framework linking peripheral metabolic activity to central nervous system health. The traditional view of lactate as metabolic waste has shifted substantially; it is now recognized as an inter-organ energy carrier, a signaling molecule with broad physiological effects, and a potential epigenetic regulator through histone lactylation. This review explores how regular exercise driven lactate pulses form a metabolic bridge linking muscle pressure with brain adaptation. This signal crosses the BBB through MCT and exerts a dual protective effect in the brain: on the one hand, it serves as a favorable energy substrate for neurons, encouraging high demand neural activity; On the other hand, it establishes cognitive resilience and neuroplasticity through multidimensional processes, including activating the GPR81 receptor signaling pathway, inducing histone acetylation modification, upregulating BDNF expression, regulating neurovascular coupling, and inhibiting neuroinflammation induced by microglia. However, the aging process can lead to systematic and multifaceted failures of this complex signaling cascade: (1) skeletal muscle atrophy resulting in impaired signal output; (2) Dysfunction of the circulatory system and blood-brain barrier hinders signal transmission; (3) The metabolic changes of astrocytes, reduced effectiveness of GPR81 receptor signaling transduction, and impaired epigenetic reactivity in the brain collectively lead to weakened signal reception and response. This functional decline in the entire production transmission response chain not only damages the natural protective effect of exercise on the brain, but also triggers a vicious cycle of neuromuscular co aging, which is closely related to the pathological development of NDs such as Alzheimer’s disease.

Based on these conclusions, future evidence and practice should further explore several core viewpoints to expand the scope from theoretical understanding to translation applications. Future evidence needs to focus on multi-level biomarkers that can quantify the functional status of the lactate signaling axis, such as dynamic lactate profiles, MCT/GPR81 expression imaging, and specific histone lactate levels. By combining individual genotypes, metabolic phenotypes, and aging stages, predictive models can be constructed to achieve precise customization of exercise prescriptions, nutritional supplements, and drug interventions, increasing both risks and advantages. A single intervention approach may not be sufficient to completely repair the complex signaling network that is disrupted during the aging process. Future efforts should investigate the synergistic effects of integrated behavioral, pharmacological, and nutritional strategies. For example, it is possible to explore combining precise exercise programs that improve lactate signaling with GPR81 allosteric modulators or safe lactate precursor supplements to determine whether they have additive or synergistic effects in promoting brain metabolism, increasing neuroplasticity, and delaying cognitive decline. The effect of lactate is significantly dependent on the environment, and there may be significant differences between acute cerebral ischemia and chronic neurodegenerative diseases. Future evidence must use spatiotemporal resolution techniques to examine the precise signal flux and functional outcomes of lactate at cell specific levels at different stages of aging and disease. This is crucial for advancing situational intelligence therapy, such as GPR81 targeted therapy activated only in specific pathological microenvironments. Well-designed clinical studies are needed to clarify the safety, feasibility, and efficacy of targeting lactate-related pathways (e.g. through specific exercise regimens, GPR81 agonists) in delaying age-related cognitive decline, promoting function in patients with sarcopenia, and as an adjuvant therapy for early ND. At the same time, the concept of maintaining a healthy lactate signaling axis should be included in public health guidelines to provide new metabolic science based reasons for improving population health and aging.
